# Purine Synthesis Inhibitor L-Alanosine Impairs Mitochondrial Function and Stemness of Brain Tumor Initiating Cells

**DOI:** 10.3390/biomedicines10040751

**Published:** 2022-03-23

**Authors:** Simranjit X. Singh, Rui Yang, Kristen Roso, Landon J. Hansen, Changzheng Du, Lee H. Chen, Paula K. Greer, Christopher J. Pirozzi, Yiping He

**Affiliations:** 1 The Preston Robert Tisch Brain Tumor Center, Duke University Medical Center, Durham, NC 27710, USA; simranjit.singh@duke.edu (S.X.S.); rui.yang@duke.edu (R.Y.); kristen.brooks@duke.edu (K.R.); landon.hansen@jhmi.edu (L.J.H.); changzheng.du@duke.edu (C.D.); lee.chen@duke.edu (L.H.C.); paula.greer@duke.edu (P.K.G.); christopher.pirozzi@duke.edu (C.J.P.); 2 Department of Pathology, Duke University Medical Center, Durham, NC 27710, USA; 3 Pathology Graduate Program, Duke University Medical Center, Durham, NC 27710, USA

**Keywords:** alanosine, adenine, purine, MTAP, glioma stem cells, mitochondria, drug resistance

## Abstract

Glioblastoma (GBM) is a lethal brain cancer exhibiting high levels of drug resistance, a feature partially imparted by tumor cell stemness. Recent work shows that homozygous *MTAP* deletion, a genetic alteration occurring in about half of all GBMs, promotes stemness in GBM cells. Exploiting MTAP loss-conferred deficiency in purine salvage, we demonstrate that purine blockade via treatment with L-Alanosine (ALA), an inhibitor of de novo purine synthesis, attenuates stemness of *MTAP*-deficient GBM cells. This ALA-induced reduction in stemness is mediated in part by compromised mitochondrial function, highlighted by ALA-induced elimination of mitochondrial spare respiratory capacity. Notably, these effects of ALA are apparent even when the treatment was transient and with a low dose. Finally, in agreement with diminished stemness and compromised mitochondrial function, we show that ALA sensitizes GBM cells to temozolomide (TMZ) in vitro and in an orthotopic GBM model. Collectively, these results identify purine supply as an essential component in maintaining mitochondrial function in GBM cells and highlight a critical role of mitochondrial function in sustaining GBM stemness. We propose that purine synthesis inhibition can be beneficial in combination with the standard of care for *MTAP*-deficient GBMs, and that it may be feasible to achieve this benefit without inflicting major toxicity.

## 1. Introduction

Glioblastoma (GBM) is the most prevalent and lethal primary brain tumor [1]. GBM patients have poor overall survival, owed in part to GBM’s heterogeneous and drug resistant nature [2]. Heterogeneity and drug resistance in GBM is partly driven by the presence of brain tumor initiating cells (BTICs), also known as GBM stem-like cells (GSCs) [3,4,5,6], as supported by these cells’ superior resistance to radiation and chemotherapy [3,5,7]. Recent studies have provided critical insights into the genesis and maintenance of BTICs and offered promising targeting strategies, including epigenetic and metabolic approaches [8,9,10,11].

Our recent study has shown that homozygous deletion of *MTAP* (methylthioadenosine phosphorylase, located on chromosome 9p21), a genetic alteration occurring in about 45% of GBMs, promotes the stemness of GBM cells and leads to more aggressive tumors [12]. MTAP functions in the adenine salvage pathway, and loss of this enzyme renders cells sensitive to purine deprivation [12,13]. In preclinical models, pharmacological inhibition of de novo purine synthesis was efficacious as a monotherapy against *MTAP*-deficient GBM and reduced the percentage of GBM cells positive for CD133, a glioma stem cell marker [4,12]. However, the role of purine supply in maintaining stemness in *MTAP*-deficient GBMs and the underlying cellular effects of purine synthesis inhibition remain unclear.

In addition, a potential strategy for therapeutically depriving purine supply in *MTAP*-deficient tumor cells is to target enzymes in the de novo purine synthesis pathway. This strategy is exemplified by the utilization of L-Alanosine (ALA), an antimetabolite that inhibits adenylosuccinate synthetase (ADSS), an enzyme which performs the penultimate step in adenosine synthesis [14,15]. Notably, while this strategy of exploiting *MTAP* loss for treatment is feasible in principle, as shown by our previous study using preclinical in vivo GBM models [12], a previous clinical trial including several other cancer types suggests that mitigating the toxicity of this strategy, such as by implementing a less intensive treatment regimen, is necessary for its clinical application [15].

In this study, we attempt to address the two problems above. Using patient-derived *MTAP*-deficient primary GBM cultures grown as BTICs, we show that ALA attenuates GBM stemness. We demonstrate that this effect is partially mediated by attenuated mitochondrial respiration and mitochondrial spare respiratory capacity. Most notably, we reveal that these effects of ALA can be achieved even when BTICs were exposed to ALA at sub-lethal doses (i.e., doses that do not inflict acute toxicity in tumor cells), suggesting a long-lasting BTIC suppressive effect of this strategy. Finally, we show that ALA treatment sensitizes *MTAP*-deficient GBM cells to temozolomide (TMZ) in vitro and in orthotopic tumors. Collectively, this study provides insights into the critical roles of purine supply in maintaining GBM stemness, the crucial roles of mitochondria in mediating this purine dependency, and suggests a targeted strategy that can potentially improve standard-of-care GBM treatment without inflicting major toxicity.

## 2. Materials and Methods

### 2.1. Cell Culture

Primary GBM cells were derived and cultured as previously described [12]. Briefly, GBM 12-0160, GBM 12-0106, and GBM 12-0358 cultures were tumor samples obtained by the Duke University Brain Tumor Center (Durham, NC, USA) and were maintained in human neural stem cell (NSC) medium (STEMCELL, Vancouver, BC, Canada; cat# 05751) supplemented with EGF 20 ng/mL, FGF 10 ng/mL, and heparin 2 µg/mL. These cells were plated and passaged on laminin-coated tissue culture plates. Transformation of normal human astrocytes was performed as previously described [12,16]. Briefly, cells were transduced with four defined genetic factors (*OCT4*, *MYC* (T58A), *HRAS* (G12V), and *TP53* dominant negative) and cultured in NSC medium [12]. Seven to ten days after transduction, cells were either transduced again with CRISPR/Cas9 lentivirus and sgRNAs targeting *MTAP* (Transformed Astrocyte #6 [12]) or treated constitutively with MTAP inhibitor, methylthio-DADMe-Immucillin A (MTDIA) [17] (Transformed Astrocyte #1). CRISPR/Cas9 targeting and MTDIA treatment were performed as in Hansen et al. [12]. All cell lines were maintained in a humidified incubator at 37 °C with 5% CO_2_.

### 2.2. Extreme Limiting Dilution Assay

Sphere forming capacity of GBM cells was assessed using the Extreme Limiting Dilution Assay (ELDA). Decreasing numbers of cells per well (33, 11, 3, and 1; 12 wells per condition) were seeded into 96 well flat-bottom plates in NSC medium with or without ALA. In the pretreatment experiment, GBM suspension tumor spheres were treated with ALA (0.25 µM) for two weeks in NSC medium in 10 cm plates prior to being brought into single cell suspension with Accutase (Innovative Cell Tech, San Diego, CA, USA; cat# AT-104) and seeded for sphere formation. Two to three weeks after plating, the presence and number of tumor spheres in each well were recorded. ELDA analysis was conducted using the online ELDA software (http://bioinf.wehi.edu.au/software/elda, accessed on 1 January 2021) [18].

### 2.3. Transcriptomic Analysis: mRNA-seq

GBM 12-0160 and GBM 12-0106 cells were treated with vehicle or ALA (0.25 µM) for 2 weeks, after which total RNA was extracted using the Quick-RNA Miniprep kit (Zymo Research, Irving, CA, USA; cat# R1054). Paired-end 150 bp sequencing was performed by Novogene on an Illumina NovaSeq 6000 machine (San Diego, CA, USA). Data quality control performed by Novogene; Bowtie 2 was used for alignment and Cufflinks 2.2.2 was used for gene expression profiling. HTSeq and edgeR were used for differential count analysis. For differential expression analysis, limma was used and cell lines were pooled based on treatment condition. Genes with FPKM values of at least 10 were included. Processed mRNA-seq data are available in Tables S1 and S2. Gene set enrichment analysis (GSEA)-associated data are available in Table S3.

### 2.4. mtDNA Abundance Analysis

GBM 12-0160 and GBM 12-0106 cells were treated with vehicle or ALA (0.25 µM or 0.5 µM) for 2 weeks, after which total DNA was extracted using the QIAamp DNA mini kit (QIAGEN, cat# 51304). mtDNA-specific primers (*MT-ND3*_F: ACACCCTCCTAGCCTTACTAC, *MT-ND3*_R: GATATAGGGTCGAAGCCGC; *MT-ND4*_F: CCTGACTCCTACCCCTCACA, *MT-ND4*_R: ATCGGGTGATGATAGCCAAG) were used to amplify mtDNA and *ACTB* primers (*ACTB*_F: AAGATGACCCAGGTGAGTGG, *ACTB*_R: AACGGCAGAAGAGAGAACCA) were used to amplify genomic DNA. Quantitative PCR was performed on a Bio-Rad CFX PCR machine and relative abundance of mtDNA was determined by normalizing mtDNA genomic material (*MT-ND3*, *MT-ND4*) abundance to nuclear genomic material (*ACTB*) abundance.

### 2.5. In Vitro Proliferation and Apoptosis Assays

Patient-derived GBM cells were plated in laminin-coated 96 well plates and Transformed Astrocytes were plated in suspension in 96 well plates. All cells were plated in NSC medium with different concentrations of TMZ (MedKoo, Morrisville, NC, USA; cat# 100810) and/or ALA (MedKoo, cat# 200130), or CPI-613 (Cayman Chemical, Ann Arbor, MI, USA; cat# 16981), or Luteolin (Tocris, Bristol, UK; cat# 2874). Proliferation was assayed by Cell Counting Kit-8 (Dojindo, Rockville, MD, USA; cat# CK04-20) or by Incucyte Live Cell Imaging system (Essen BioScience, Ann Arbor, MI, USA) as indicated in figure legends. Apoptosis/Annexin V measurement was performed using the Incucyte Annexin V Green reagent (Essen BioSciences, cat# 4642) in conjunction with the Incucyte Live Cell Imagining system, as recommended by the manufacturer. Lengths of proliferation assays are indicated in figure legends. We note that the potency of ALA from MedKoo (or other available sources) varied significantly and/or from batch to batch. In this study, batches of ALA with comparable potency, as tested by in vitro proliferation assays, were used.

### 2.6. Seahorse XF Analysis

Metabolic flux assays measuring oxygen consumption rate (OCR) and extracellular acidification rate (ECAR) were performed using the Seahorse XFe96 Analyzer (Agilent, Santa Clara, CA, USA). Patient-derived GBM cells were seeded at a density of 1 to 1.5 × 10^4^ cells per well in NSC medium in laminin-coated Seahorse XFe96 cell culture microplates. The plate was incubated at 37 °C for several hours to allow cells to adhere. Different concentrations of ALA, TMZ, or CPI-613 diluted in NSC medium were added to the wells and incubated for 24 h at 37 °C. The following day, the medium was replaced with Seahorse Phenol Red-free DMEM containing appropriate dilutions of ALA, TMZ, or CPI-613. The plate was incubated in a CO_2_-free 37 °C incubator for 1 h and a Mitochondrial Stress Test (Agilent) was performed according to manufacturer’s instructions. Following the mitochondrial stress test, the plate was imaged in an Incucyte Live Cell Imaging system (Essen BioScience). Cell confluence was quantified for each well and used for normalizing OCR and ECAR measurements.

### 2.7. Synergy/Antagonism Analysis

To determine possible synergistic or antagonistic effects of TMZ and ALA combination treatment, proliferation data from the Incucyte Live Cell Imaging system were analyzed using Combenefit software, version 2.021 (Cambridge, UK) (www.cruk.cam.ac.uk/research208groups/jodrell-group/combenefit, accessed on 1 January 2021) [19]. The synergy/antagonism score for each combination was calculated by Combenefit using the Loewe synergy model, where a score > 0 indicates synergy, a score of 0 indicates additive effects, and a score < 0 indicates antagonism.

### 2.8. In Vivo Drug Response Studies

Orthotopic intracranial tumors were generated by injecting cells (GBM 12-0160, expressing luciferase) into the right caudate nucleus of female athymic nude mice (Jackson Labs, Bar Harbor, ME, USA; stock# 007850). Cells were mixed 1:3 with methylcellulose and a total of 1 × 10^5^ cells were implanted into each animal. Tumors were allowed to form for 4 weeks after implantation. To establish baseline tumor bioluminescence, mice were injected with D-luciferin 15 mg/kg (GoldBio, St. Louis, MO, USA; cat# LUCNA) and scanned in an IVIS Lumina XR imager. Mice were then randomized into four groups and drug treatment initiated. Mice received three intraperitoneal (IP) injections of TMZ 5 mg/kg (MedKoo, cat# 100810) during the first week of treatment followed by three weeks off, representing one cycle of TMZ treatment. Mice received three IP injections of ALA 150 mg/kg (MedKoo, cat# 200130) weekly for the duration of the treatment period. Saline IP injections were used as the vehicle control treatment. In vivo drug response was monitored by weekly bioluminescent imaging of mice as described above and analyzed using Living Image software. All animal experiments were performed in accordance with protocols approved by the Duke University Institutional Animal Care and Use Committee (Duke IACUC; Durham, NC, USA).

### 2.9. Statistics

All in vitro experiments in this study were performed independently at least twice. Data presented as mean +/− standard error of the mean (SEM). Mean values between two groups were compared using *t*-tests (two-tailed, unpaired, with Welch’s correction) when data were assumed to follow a normal distribution, otherwise non-parametric Mann-Whitney test was performed. Multiple groups were compared using ANOVA, followed by Tukey’s or Sidak’s multiple comparisons tests. Statistical test results were deemed significant if *p* < 0.05. With the exception of ELDA experiments and Combenefit synergy analysis, all statistical analyses were calculated using Graphpad Prism software, version 9.3.1 (San Diego, CA USA).

## 3. Results

**Figure 1:**
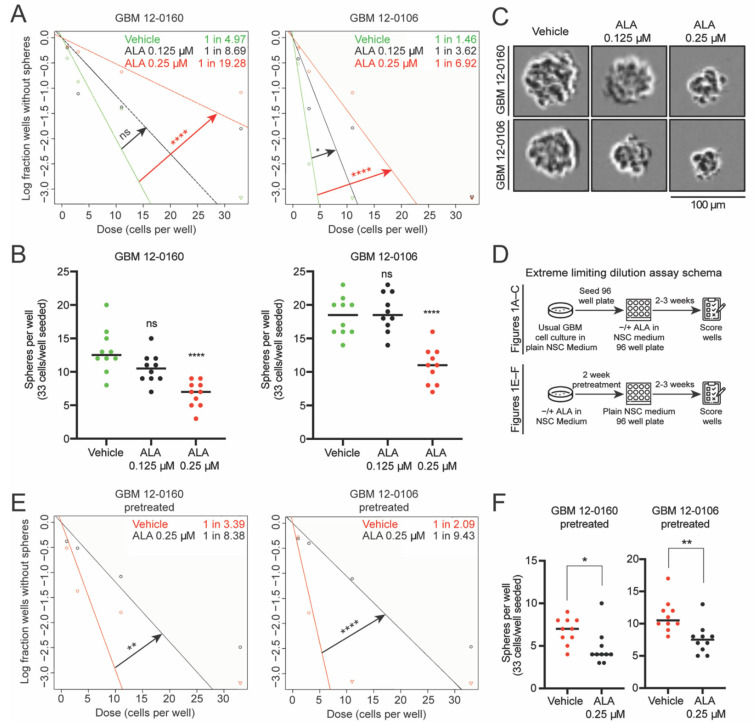
Inhibition of de novo purine synthesis using L-Alanosine (ALA) decreases stem cell frequency and sphere-forming capacity of *MTAP*-deficient glioblastoma (GBM) cells. (**A**) Extreme limiting dilution assays (ELDAs) show ALA treatment decreases stem cell frequency in two *MTAP*-deficient patient-derived GBM cell lines. Stem cell frequency (1 in n) for each condition shown on plot. 33, 11, 3, 1 cells seeded per well, *n* = 12 replicates per condition. (**B**) ALA treatment decreases the number of spheres formed per well in ELDA. 33 cells seeded per well, *n* = 10 replicates per condition. (**C**) Representative images of tumor spheres derived from GBM 12-0160 and GBM 12-0106. Scale bar, 100 μm. (**D**) Schema depicting design of ELDAs. Assays shown in (**A**–**C**) done with concurrent ALA treatment in 96 well plates; assays shown in (**E**,**F**) done with 2 weeks ALA pretreatment followed by seeding cells in neural stem cell (NSC) medium without ALA in 96 well plates. (**E**) Extreme limiting dilution assays (ELDAs) show 2-week pretreatment with ALA decreases stem cell frequency in GBM cells. Stem cell frequency (1 in *n*) for each condition shown on plot. 33, 11, 3, 1 cells seeded per well, *n* = 12 replicates per condition. (**F**) 2-week pretreatment with ALA decreases the number of spheres formed per well in ELDA. 33 cells seeded per well, *n* = 10 replicates per condition. Data shown are mean +/− SEM. Data analyzed using ELDA Chi-square test (**A** and **E**), One-Way ANOVA followed by multiple *t*-tests (**B**,**F**); ns: not significant, * *p* < 0.05, ** *p* < 0.01, **** *p* < 0.0001.

To investigate the role of purine supply in maintaining stemness, we employed naturally *MTAP*-deficient patient-derived GBM cell lines (GBM 12-0160 and GBM 12-0106), which were cultured in neural stem cell (NSC) medium to maintain their BTIC properties and were previously shown to be susceptible to ALA treatment [12]. We subjected these cell lines to extreme limiting dilution assay (ELDA) analysis [18], seeding 33, 11, 3, and 1 cell per well, in the presence of 0.125 µM or 0.25 µM ALA. These doses exhibited no or minimal effects on proliferation, which ensured the absence of acute toxicity and the confounding variable of cell growth inhibition in assessing changes in stemness (Figure S1A). Overall, the ELDAs demonstrate that ALA treatment leads to a dose-dependent reduction in stem cell frequency (Figure 1A). Furthermore, ALA treatments (at 0.25 µM) significantly reduce the number of spheres formed per well (Figure 1B), and reduce tumor sphere size (Figure 1C and Figure S1B). In contrast, we found that ALA treatment has no effect on sphere formation capacity when performing the ELDA with an *MTAP*-wildtype (WT) patient-derived GBM cell line (Figure S1D), supporting the specific effect of this treatment on *MTAP*-deficient tumor cells. Since we focus on *MTAP*-deficient GBMs, we do not include this *MTAP*-WT cell line for further investigation. Additionally, although we cannot rule out the effects of other genetic alterations on ALA response, these results are consistent with our previous findings that *MTAP* genotype is likely one factor [12]. Collectively, these results demonstrate that the presence of ALA during the sphere-forming period (two to three weeks) decreases stem cell frequency in the *MTAP*-deficient primary GBM cultures.

Next, we determined whether ALA’s suppressive effects on stemness are dependent on its constant presence in the media. This is important, as a long lasting suppressive effect makes it more likely that a short period of ALA exposure can be therapeutically beneficial. We pretreated GBM cells for two weeks with vehicle or with ALA (0.25 µM), and subsequently seeded the pretreated cells in NSC medium free of ALA for ELDA (Figure 1D). Remarkably, we found that GBM cells pretreated with ALA also display a reduction in stem cell frequency (Figure 1E). Furthermore, this reduced sphere-formation frequency is supported by the decreased numbers of spheres in wells where sphere-formation occurred (Figure 1F), and by reduced sphere size (Figure S1C). Together, these results suggest that pretreatment with ALA (i.e., transient exposure to purine shortage) confers a sustained diminishment of stemness in GBM cells.

**Figure 2:**
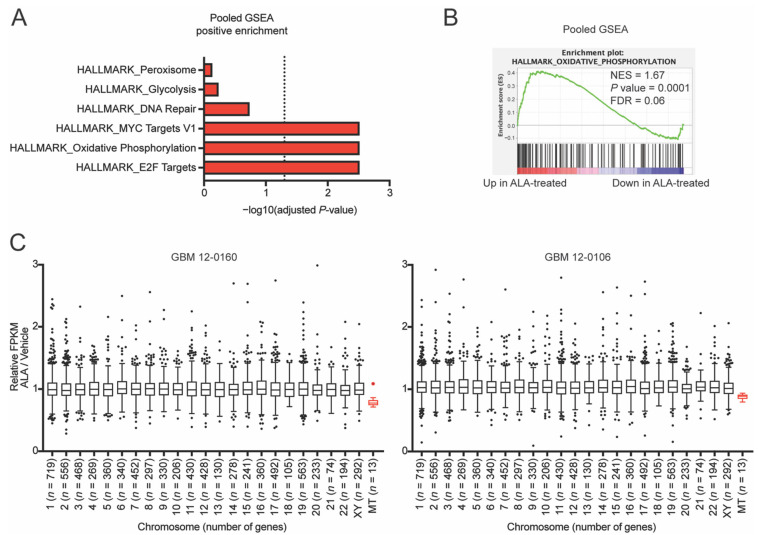
Inhibition of de novo purine synthesis using L-Alanosine (ALA) alters glioblastoma (GBM) cells’ oxidative phosphorylation genes and mtDNA gene expression. (**A**) Gene set enrichment analysis (GSEA) of pooled mRNA-seq of GBM 12-0160 and GBM 12-0106 cells pretreated for 2 weeks with ALA 0.25 µM highlights positively enriched Hallmark pathways; dashed line represents *p* = 0.05. (**B**) GSEA enrichment plots for oxidative phosphorylation show a positive enrichment with ALA treatment. (**C**) Individually analyzed mRNA-seq shows that ALA decreases expression of mitochondrial DNA-encoded (MT) genes (shown in red). Genes with FPKM of at least 10 were included in analysis. Outlier genes (*n* = 3 genes) excluded from plot for data visualization purposes. *p* < 0.0001 for MT vs. each other chromosome. Data shown are mean +/− SEM. Data analyzed using Mann-Whitney test (**C**).

To investigate the underlying cellular impacts of the exposure to ALA, we treated GBM cell lines with ALA (at 0.25 µM) for two weeks, and performed mRNA-sequencing to determine the changes in the transcriptomic profile (Table S1). Next, we pooled the cell lines based on treatment condition (ALA-treated vs. vehicle-treated) and conducted differential expression analysis (Table S2). Using this pooled dataset, we performed gene set enrichment analysis (GSEA) [20], and found that the oxidative phosphorylation pathway genes are positively enriched (Figure 2A,B and Table S3). Performing GSEA on the two cell lines individually also highlights a positively enriched oxidative phosphorylation gene set (Figure S2A,B). We speculated that the ALA-induced positively enriched oxidative phosphorylation pathway may indicate either (i) enhanced mitochondrial function, which is less likely as this pathway’s genes are overwhelmingly encoded by the nuclear genome instead of the mitochondrial genome, or (ii) compensatory feedback due to weakened mitochondrial function. This latter possibility is supported by the following independent lines of evidence.

First, when assessing mitochondrial DNA (mtDNA)-specific genes, we found that ALA treatment results in an approximately 20% reduction in transcriptional output from mtDNA in comparison to the vehicle-treated cells, in contrast to the mostly unaffected output from nuclear chromosomes (Figure 2C). PCR quantification of the mtDNA showed that mtDNA copy number is unaffected by ALA treatment (Figure S2C), suggesting a deficiency in the gene transcriptional machinery in the mitochondria. Second, to further probe the effects of ALA treatment on mitochondrial function, we utilized Seahorse XF analysis to conduct a mitochondrial stress test on the GBM cells treated with ALA. Initially, we sought to test the effects of acute 24-h ALA treatment, and found that high doses of ALA (3 µM and 10 µM) moderately reduce maximal oxygen consumption rate (OCR) (Figure S3A). Furthermore, the addition of exogenous adenine to cells undergoing ALA treatment rescues this reduction in maximal OCR, suggesting this reduced OCR is due to adenine shortage, instead of off-target effects of ALA (Figure S3B).

**Figure 3:**
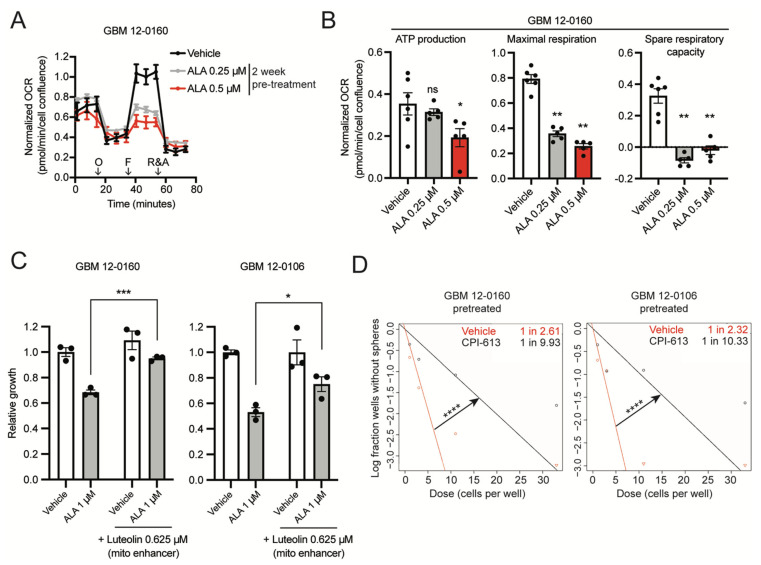
Inhibition of de novo purine synthesis using L-Alanosine (ALA) reduces glioblastoma (GBM) cells’ mitochondrial respiration and eliminates spare respiratory capacity. (**A**) Seahorse XF analysis of GBM 12-0160 pretreated for 2 weeks with ALA 0.25 µM or 0.5 µM shows ALA reduces maximal respiration. *n* = 6 replicates for Vehicle, *n* = 5 replicates for each other condition. O: Oligomycin, F: FCCP, R&A: Rotenone and Antimycin A. (**B**) Two-week pretreatment with ALA reduces ATP production, maximal respiration, and spare respiratory capacity in GBM 12-0160. *n* = 6 replicates for Vehicle, *n* = 5 replicates for each other condition. (**C**) Luteolin (mitochondria respiration enhancer) treatment at 0.625 µM partially rescues ALA-induced proliferation inhibition. Growth assayed with Incucyte Live Cell Imaging system, 5 days (percentage confluence). Growth shown relative to Vehicle condition, *n* = 3 replicates for each condition. (**D**) Extreme limiting dilution assays (ELDAs) show 2-week pretreatment with CPI-613 100 µM decreases stem cell frequency in GBM cells. Stem cell frequency (1 in *n*) for each condition shown on plot. 33, 11, 3, 1 cells seeded per well, *n* = 10 replicates per condition. Data shown are mean +/− SEM. Data analyzed using Mann-Whitney test (**B**), Welch’s *t*-test (**C**), ELDA Chi-square test (**D**); ns: not significant, * *p* < 0.05, ** *p* < 0.01, *** *p* < 0.001, **** *p* < 0.0001.

Finally, we further assessed the impact of pre-exposure to ALA on OCR. We pretreated GBM cells with low doses of ALA (0.25 µM and 0.5 µM) for two weeks, and subsequently subjected the cells to similar Seahorse assays in media free of ALA. We found that the pre-exposure to ALA results in significantly attenuated maximal OCR (Figure 3A), and eliminates mitochondrial spare respiratory capacity (Figure 3B). These results suggest that pre-exposure to ALA leads to compromised mitochondrial function; therefore, these data indicate a lasting effect of purine shortage on mitochondrial function in GBM cells, reminiscent of its enduring suppressive effects on GBM stemness. Collectively, these results suggest that exposure to purine shortage compromises mitochondrial function in BTICs.

The concurrent suppressive effects of ALA treatment on stemness and mitochondrial function of BTICs led us to hypothesize that compromised mitochondrial function mediates ALA’s suppressive effects on GBM stemness. To test this hypothesis, we utilized luteolin, a flavonoid compound that has been shown to enhance mitochondrial respiration in neuronal cells [21]. After testing a range of doses, we observed that at 0.625 µM, luteolin has no promoting or adverse effects on GBM cell proliferation (Figure S3C). We found that, indeed, 0.625 µM luteolin treatment partially rescues the inhibition of ALA-treated GBM cells (Figure 3C). To further implicate mitochondrial activity in the maintenance of GBM stemness, we employed CPI-613, a mitochondrial respiration inhibitor [22]. As expected, treatment with CPI-613 inhibits overall mitochondrial respiration in a dose-dependent manner (Figure S3D,E). At 100 µM, cell proliferation is largely unaffected, but mitochondrial respiration is inhibited (Figure S3D,E); we therefore used this dose for the following ELDA experiments. We pretreated GBM cells with vehicle or with CPI-613 for two weeks and subjected them to ELDA in NSC medium (without CPI-613). ELDA analysis showed that CPI-613 pretreatment (100 µM) attenuates stem cell frequency, recapitulating the stemness reduction seen in ALA-pretreated cells (Figure 1E and Figure 3D). Together, these results reveal the critical roles of mitochondrial function in the maintenance of BTICs, and suggest compromised mitochondrial function at least partially mediates the diminished GBM stemness caused by purine shortage.

A striking result of the Seahorse XF analyses, shown in Figure 3A,B, is that ALA treatment eliminates GBM cells’ spare respiratory capacity (also known as reserve respiratory capacity), a measure of cellular fitness [23,24]. Higher spare respiratory capacity has been associated with radioresistance, resistance to cell death, and lower production of reactive oxygen species (ROS) [23,24,25]. Furthermore, GBM cells that acquired TMZ resistance were found to display a higher spare respiratory capacity, suggesting the importance of mitochondrial reserve in GBM cells’ response to TMZ [26]. Collectively, these previous findings, together with the weakened stemness and elimination of spare respiratory capacity by ALA, led us to ask whether ALA treatment can sensitize *MTAP*-deficient GBM cells to TMZ, a standard of care drug for GBMs.

**Figure 4:**
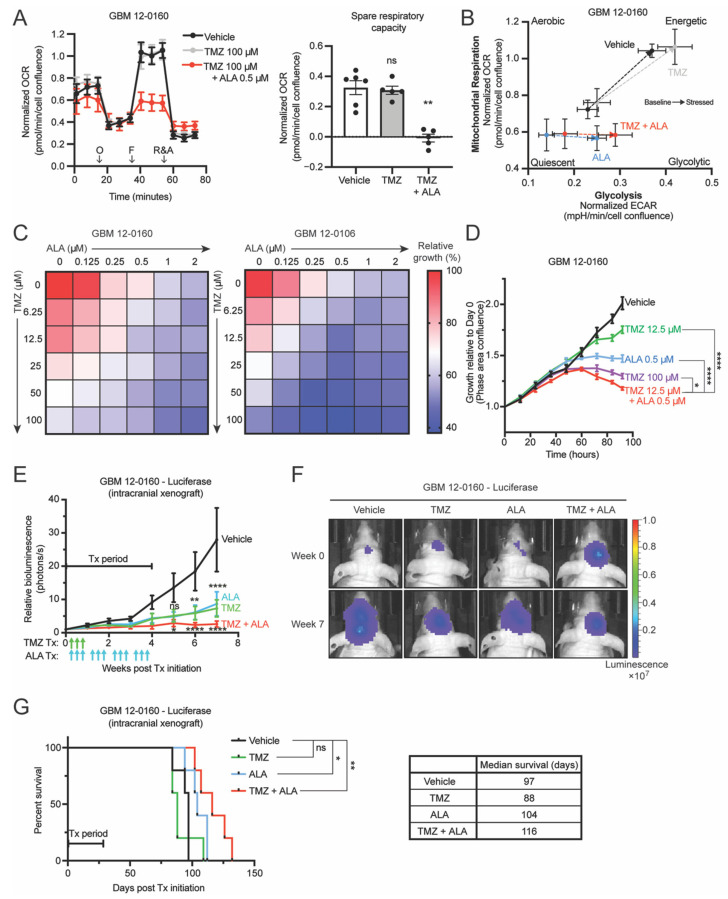
Inhibition of de novo purine synthesis using L-Alanosine (ALA) attenuates *MTAP*-deficient glioblastoma (GBM) cells’ resistance to TMZ in vitro and in vivo. (**A**) Seahorse XF analysis of GBM 12-0160 shows that 2-week ALA 0.5 µM-pretreated cells maintain attenuated maximal respiration (left) and abolished spare respiratory capacity (right) when treated acutely (24 h) with TMZ. *n* = 6 replicates for Vehicle, *n* = 5 replicates for each other condition. O: Oligomycin, F: FCCP, R&A: Rotenone and Antimycin A. (**B**) Seahorse XF cell energy phenotype analysis shows that ALA 0.5 µM-pretreated cells (ALA alone and TMZ + ALA) are unable to shift their metabolism toward an “energetic” phenotype to meet induced energy demands. Arrows indicate Baseline to Stressed condition. Stressor compounds: oligomycin (ECAR) and FCCP (OCR). (**C**) Heatmaps show combination effect of TMZ and ALA on growth of two *MTAP*-deficient GBM cell lines. Growth assayed with Incucyte Live Cell Imaging system, 4 days (percentage confluence). Growth shown relative to Vehicle condition, *n* = 3 replicates for each drug combination. (**D**) Longitudinal proliferation assay shows low-dose combination TMZ + ALA treatment is more efficacious than single agent treatment and as efficacious as high-dose TMZ alone in GBM-120160. Growth assayed with Incucyte Live Cell Imaging system (percentage confluence). Growth shown relative to time 0 for each treatment condition, *n* = 3 replicates. (**E**) Intracranial xenograft of GBM 12-0160–Luciferase in nude mice given either vehicle, single agent, or combination treatments. Growth curves show combination TMZ and ALA is more efficacious than single agent treatment in inhibiting GBM growth in vivo, *n* = 5 mice per group. For statistics, each group is compared to Vehicle. *p*-value summaries labeled above ALA and TMZ curves (ns, **, ****) represent significance for both ALA and TMZ curves. Tx: Treatment. Green arrows: TMZ administered; Blue arrows: ALA administered. (**F**) Representative bioluminescence images from mice in (**E**) depicting change in intracranial tumor luciferase signal from Week 0 to Week 7. (**G**) Kaplan-Meier analysis shows that combination treatment extends mouse median survival more than either single agent alone, *n* = 5 mice per group. Data shown are mean +/− SEM. Data analyzed using Mann-Whitney test (**A**, right), Two-Way ANOVA followed by Tukey’s multiple comparisons test (**D**,**E**), *p*-values labeled in (**D**) represent Tukey’s test comparison for latest time point; Log-rank test (**G**); ns: not significant, * *p* < 0.05, ** *p* < 0.01, **** *p* < 0.0001.

First, we conducted Seahorse XF analyses treating cells for 24 h with vehicle, TMZ alone, or TMZ combined with 2-week ALA pretreatment. We observed that with TMZ treatment alone (at 100 µM), for 24 h in advance and with its continuous presence in the media during the Seahorse assay, cells maintain a positive reserve capacity (Figure 4A), and that ALA-pretreated cells, when treated with TMZ in the same manner, display diminished spare respiratory capacity (Figure 4A). Interestingly, analysis of extracellular acidification rate (ECAR) showed that 2-week ALA pretreatment combined with TMZ impairs cells’ ability to increase ECAR in response to stressed conditions (oligomycin) when compared with vehicle or TMZ alone (Figure S3F). Consistent with these observations, Seahorse cell energy phenotype analysis shows that ALA-pretreated cells (ALA alone and TMZ + ALA) were unable to shift their metabolism toward an “energetic” phenotype to meet induced energy demands, reflecting diminished cell fitness (Figure 4B).

Next, using paired MTAP-intact and MTAP-deficient cell lines, we established that MTAP-deficient GBM cells display increased resistance to TMZ (Figure S4A,B), agreeing with our previous finding [12]. The need for a strategy to overcome this resistance, together with the aforementioned findings, prompted us to hypothesize that imposing purine shortage can be used to sensitize BTICs to TMZ. To test this hypothesis, we subjected BTICs to various combinations of TMZ and ALA in vitro. We used the Incucyte Live Cell Imaging system to track proliferation of cells and assess the efficacy of combination treatments. Indeed, we observed synergistic suppressive effects of these two agents (Figure S5A,B), and found that several combinations of TMZ and ALA are more efficacious in inhibiting BTIC propagation than either TMZ or ALA alone (Figure 4C). Notably, we observed that a low dose (12.5 µM) of TMZ in the presence of a low dose (0.5 µM) of ALA displays efficacy comparable to that of high dose (100 µM) TMZ alone (Figure 4C,D). In agreement with these results, imaging and longitudinal Annexin V apoptosis assays revealed that the presence of ALA (at 0.25 µM) significantly potentiates TMZ’s cytotoxicity against BTICs (Figure S5C,D). Collectively, these results echo the suppressive effects of ALA treatment on mitochondrial spare respiratory capacity and stemness of GBM cells, and suggest that ALA can be used to sensitize GBM cells to TMZ.

To further test the effect of TMZ and ALA combination treatment in vivo, we orthotopically implanted *MTAP*-deficient GBM cells expressing luciferase into athymic nude mice, randomized the mice into four groups of treatment: vehicle (saline), TMZ (5 mg/kg), ALA (150 mg/kg, a dose that was 33% lower than that was used in our previous study [12]), and TMZ + ALA (TMZ 5 mg/kg + ALA 150 mg/kg), and monitored tumor progression by weekly bioluminescent IVIS imaging (Figure S5E). We observed that single agent treatment, either TMZ or ALA alone, has moderate efficacy in vivo compared to the vehicle-treated group (Figure 4E,F). Importantly, tumors in the combination treatment group remain stable after the treatment was ceased in comparison to those treated with a single agent (Figure 4E,F). Furthermore, combination treatment extends mouse median survival more than either single agent alone (Figure 4G). Collectively, these results suggest that combining TMZ and ALA leads to a more durable response than either TMZ or ALA alone in inhibiting the progression of *MTAP*-deficient GBM in vivo.

## 4. Discussion

In this study, we exploited the susceptibility of *MTAP*-deficient GBM cells to de novo purine synthesis inhibition and examined the effects of purine blockade on the maintenance of GBMs’ stemness, mitochondrial function, and response to chemotherapy. Findings from this study advance our understanding of these critical aspects of GBMs in several ways. First, recent work has identified numerous epigenetic and metabolic factors as essential for promoting GBM stemness and consequently as worthwhile therapeutic targets [8,9,10,11,27,28,29]. In particular, it has been shown that the guanine synthesis arm of purine metabolism can be targeted for overcoming GBM stemness [30]. Resonating with and complementary to this previous study [27], our finding that purine/adenine shortage can attenuate the stemness of GBMs illustrates a critical role of the adenine synthesis arm of purine metabolism in promoting GBM stemness and establishes adenine synthesis as an additional therapeutic vulnerability for GBMs. This GBM cell-specific purine blockade strategy is facilitated by the fact that about half of GBMs bear homozygous *MTAP* deletions, making them deficient in adenine salvage and uniquely susceptible to this therapeutic strategy. Moreover, the suppressive effect of transient purine synthesis inhibition on GBM stemness further supports the feasibility of this therapeutic strategy.

Second, through transcriptomic and metabolic analyses, we provide direct evidence to support the essential role of mitochondria in maintaining GBM stemness and suggest that attenuated mitochondrial respiration and spare respiratory capacity partially underlie the effects of purine blockade on the proliferation and stemness of BTICs. These findings echo the distinct metabolic profiles in BTICs in contrast to those in non-stem GBM cells [24], and support the pathogenic significance of mitochondrial function in maintaining GBM stemness. While the transcriptional output from the mtDNA in BTICs is diminished after purine shortage, we speculate that it is more likely that this is indicative rather than causative of defective mitochondrial function. The lasting effect of purine blockade on mitochondrial function in BTICs, as evidenced by measurable defects in mitochondrial function even after the blockade was removed, is particularly intriguing. Furthermore, our results are supported by previous work that shows that genetic knockout of adenylosuccinate lyase (ADSL) impairs mitochondrial function in hepatocellular carcinoma, suggesting the importance of purines for mitochondrial function [31]. Further studies illustrating the mechanistic link between purine supply and mitochondrial function, and/or a metabolic profile-based stratification of GBMs for identifying those most vulnerable to this therapeutic strategy, are needed for further potentiating purine blockade-based therapeutic strategies.

Finally, the therapeutic implication of the aforementioned findings is highlighted by purine synthesis inhibition-conferred BTIC susceptibility to TMZ, a toxic DNA-damaging agent that is currently standard-of-care for GBM patients [32]. Our GSEA did show that the DNA repair pathway is enriched in one ALA-treated GBM cell line, however further studies are required to elucidate the impact of ALA treatment on the DNA damage response. While it is possible that the effects of purine blockade on the DNA damage response contribute to the observed therapeutic benefit [33], we postulate that mitochondrial damage by purine shortage is also part of the mechanism, as the effects of purine blockade on GBM proliferation and stemness can be rescued by mitochondrial enhancer treatment and recapitulated by direct pharmacological inhibition of mitochondrial respiration. Purine blockade serves as another example to highlight the promising principle of exploiting metabolic vulnerabilities for overcoming GBM stemness and resistance to TMZ, as demonstrated by a previous study [34]. It is important to note that a previous clinical trial involving several cancer types other than GBMs revealed ALA-induced hematologic toxicities in patients [15]. Our findings suggest that a less intensive ALA regimen, such as a shorter period of treatment with a lower, less toxic dose of ALA in combination with a lower standard-of-care chemotherapy dose, may be efficacious against GBM while inflicting manageable overall toxicity. We speculate that further studies understanding the pharmacological kinetics of this agent in vivo as well as dissecting the interplay between the extent of purine shortage and the stemness of GBM will be critical for exploiting this principle and maximizing therapeutic benefit in GBM treatment.

In summary, our findings suggest that targeting purine supply attenuates stemness in *MTAP*-deficient GBM cells, and that compromised mitochondrial function underlies this effect. We propose that the major benefits of such a strategy–the attenuated GBM stemness and the sensitization to TMZ–can be leveraged for improving the clinical management of the about half of GBMs that are *MTAP*-null, potentially with tolerable toxicity.

## Data Availability

Materials used in this study are available from the corresponding author upon reasonable request. All data generated or analyzed during this study are included in this published article (and its Supplementary Information Files). Raw and processed mRNA-seq data are deposited in the Gene Expression Omnibus (GEO) repository, accession GSE197728.

## Supplementary Materials

The following supporting information can be downloaded at: https://www.mdpi.com/article/10.3390/biomedicines10040751/s1, Figure S1: Effects of ALA treatment on proliferation and sphere size in *MTAP*-deficient GBM cells and on sphere formation in *MTAP*-wildtype GBM cells; Figure S2: Effects of ALA treatment on cellular pathways and on mtDNA abundance; Figure S3: Seahorse XF analyses of ALA- or CPI-613-treated GBM cells and Luteolin dose response; Figure S4: MTAP loss facilitates TMZ resistance; Figure S5: Synergy/antagonism and apoptosis analyses of TMZ + ALA combination treatment; Table S1: Individually processed mRNA-seq data of ALA-treated patient-derived GBM cell lines; Table S2: Pooled differential expression analysis of ALA-treated patient-derived GBM cell line mRNA-seq data; Table S3: Pooled GSEA-associated data.
