# Supplementary material for: Purine Synthesis Inhibitor L-Alanosine Impairs Mitochondrial Function and Stemness of Brain Tumor Initiating Cells
Source: Biomedicines. 2022 Mar 23;10(4):751. doi: 10.3390/biomedicines10040751 (PMC9025092; doi:10.3390/biomedicines10040751)
Supplement: Supplementary file 1 [file biomedicines-10-00751-s001.zip › Singh et al 2022 Biomedicines Supplementary Figs S1-S5.pdf]

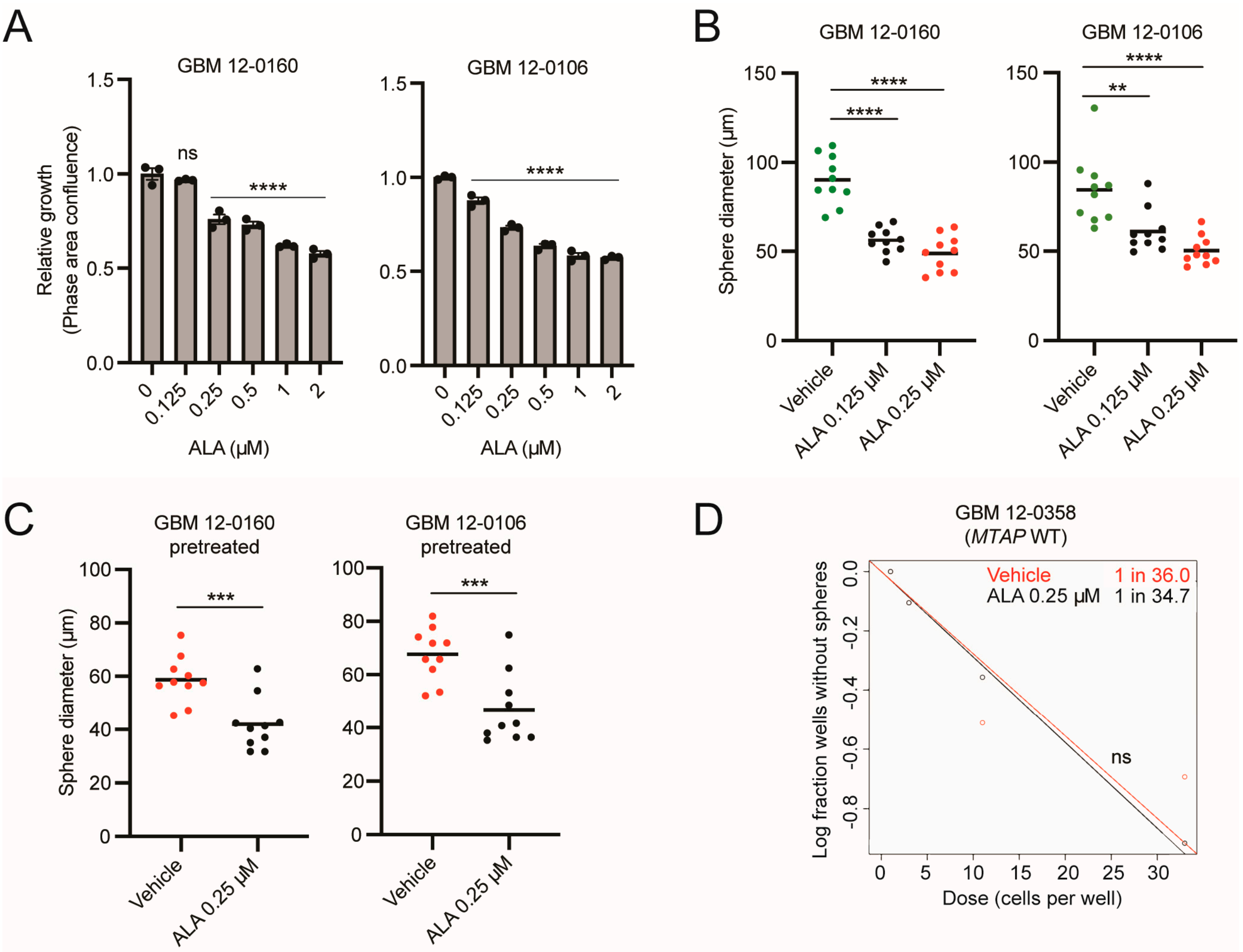

Figure S1. Effects of ALA treatment on proliferation and sphere size in *MTAP*-deficient GBM cells and on sphere formation in *MTAP*-wildtype GBM cells.

(A) Drug sensitivity plots for ALA in two GBM cell lines. Growth assayed with Incucyte Live Cell Imaging system, 4 days (confluence relative to Day 0, normalized to ALA 0 μM), n=3 replicates for each dose. **(B)** Sphere diameter quantification for Figures 1A-C shows ALA treatment decreases tumor sphere size. **(C)** Sphere diameter quantification for Figures 1E-F shows ALA pretreatment decreases tumor sphere size. **(D)** ELDA shows ALA treatment has no effect on stem cell frequency in GBM 12-0358, a *MTAP*-wildtype (WT) patient-derived GBM cell line. Stem cell frequency (1 in n) for each condition shown on plot. 33, 11, 3, 1 cells seeded per well, n=12 replicates per condition. Data shown are mean +/- SEM. Data analyzed using One-way ANOVA followed by Holm-Sidak's multiple comparisons test **(A)**, P values represent comparison of each dose to vehicle (zero); Student's t-test **(B, C)**, ELDA Chi-square test **(D)**; ns: not significant, ****$P < 0.0001$.

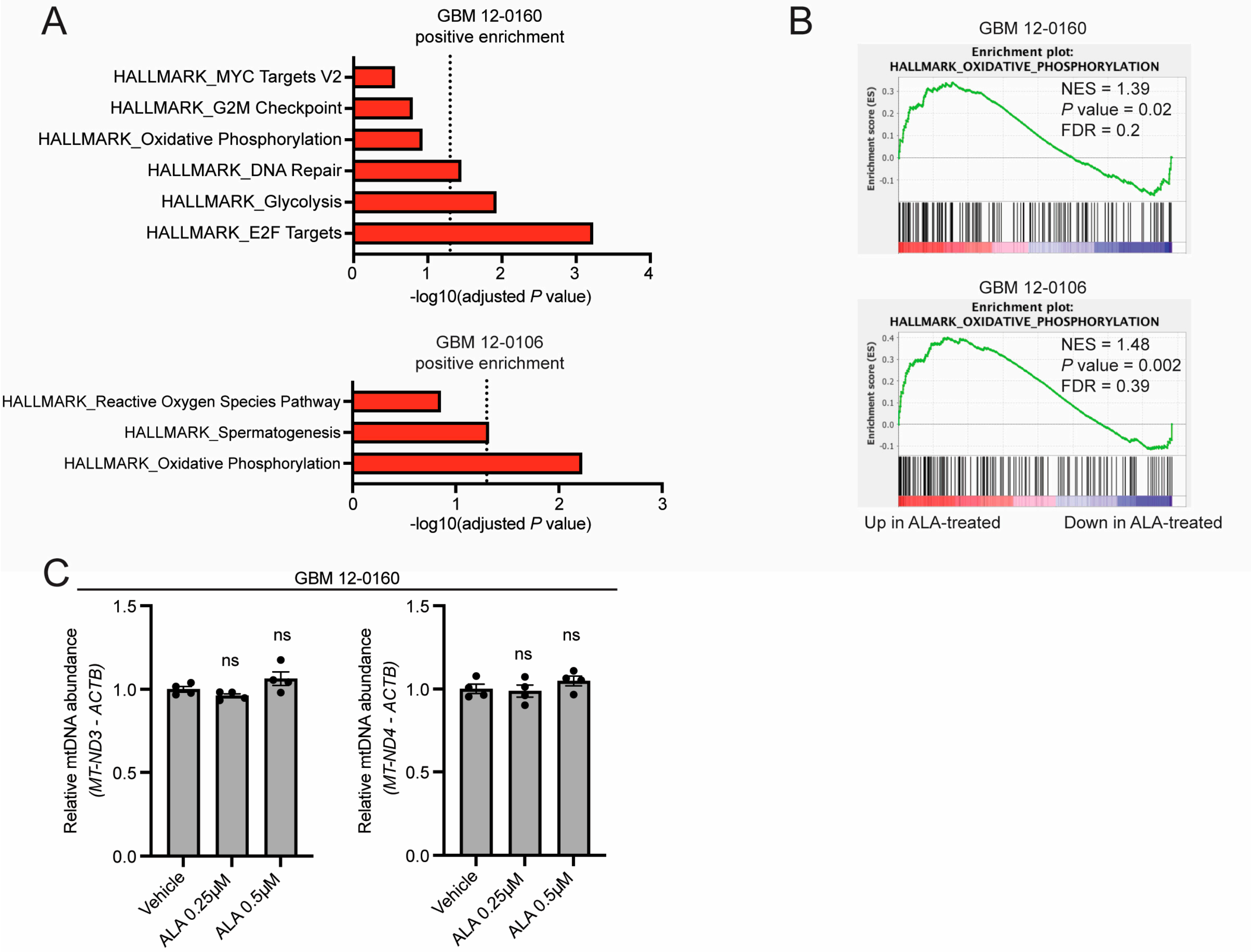

Figure S2. Effects of ALA treatment on cellular pathways and on mtDNA abundance.
(A) Gene set enrichment analysis (GSEA) of mRNA-seq of GBM 12-0160 and GBM 12-0106 cells pretreated for 2 weeks with ALA 0.25 µM highlights positively enriched Hallmark pathways; dashed line represents *P* = 0.05. **(B)** GSEA enrichment plots for oxidative phosphorylation show a positive enrichment with ALA treatment individually in each cell line. **(C)** Quantitative PCR analysis shows that mtDNA abundance is unaffected by ALA treatment (2 weeks). *MT-ND3* or *MT-ND4* amplification relative to *ACTB*, *n*=4 replicates per condition. Data shown are mean +/- SEM. Data analyzed using Mann-Whitney test **(E)**; ns: not significant.

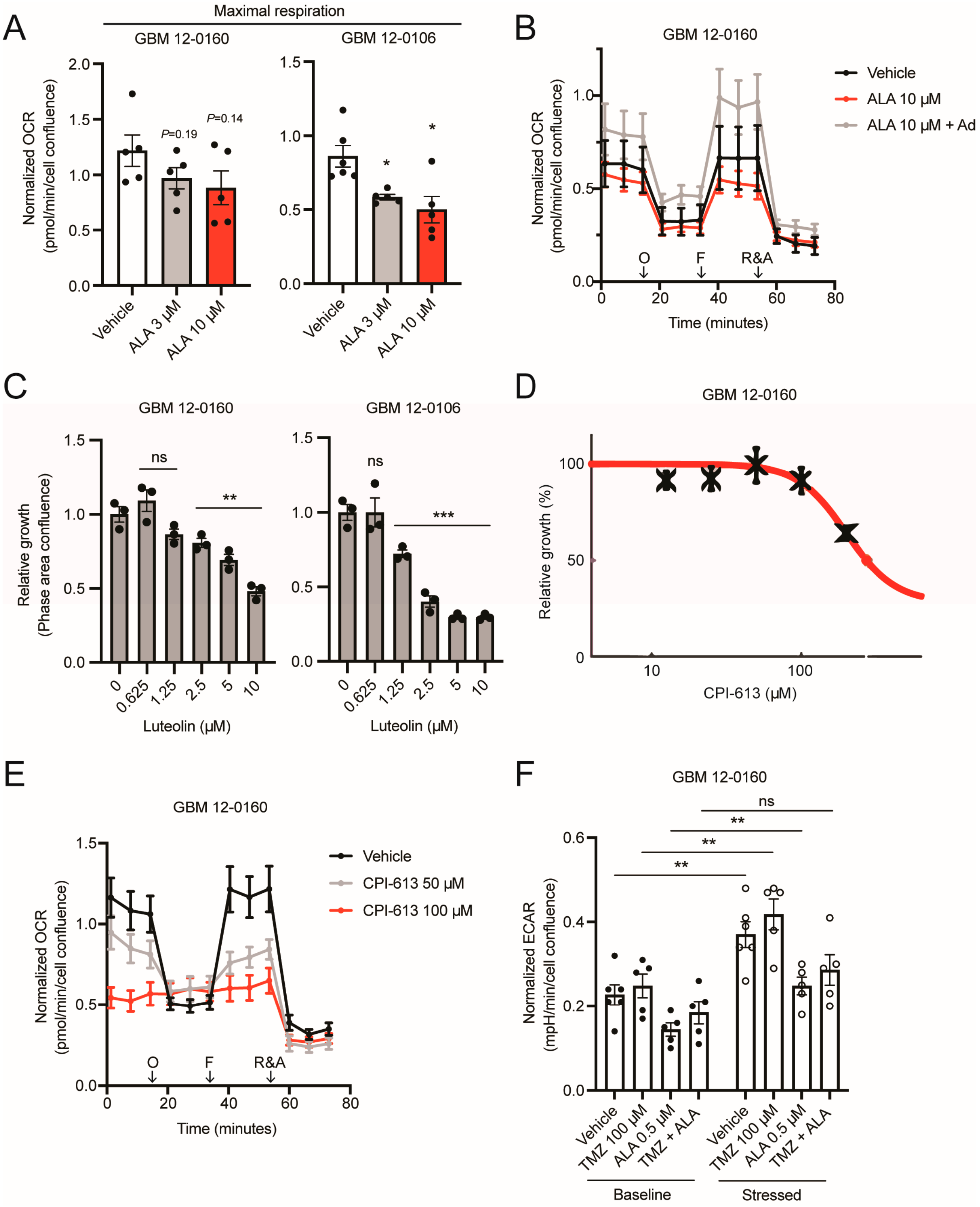

Figure S3. Seahorse XF analyses of ALA- or CPI-613-treated GBM cells and Luteolin dose response.

(A) Acute (24 hours) ALA treatment reduces maximal respiration in GBM cells. *n*=6 replicates for Vehicle (GBM 12-0106), *n*=5 replicates for each other condition. **(B)** Reduced maximal respiration in acutely (24 hours) ALA-treated cells can be rescued by Adenine (Ad) 10 µM. *n*=6 replicates for Vehicle, *n*=5 replicates for each other condition. O: Oligomycin, F: FCCP, R&A: Rotenone and Antimycin A. **(C)**

Drug sensitivity plots for Luteolin in two GBM cell lines. Growth assayed with Incucyte Live Cell Imaging system, 4 days (confluence relative to Day 0, normalized to Luteolin 0 μM), *n*=3 replicates for each dose. **(D)** CPI-613 dose response curve. Plot generated by Combenefit software, mean shown of *n*=3 replicates. **(E)** Acute (24 hour) CPI-613 treatment reduces basal and maximal respiration. *n*=6 replicates for Vehicle, *n*=5 replicates for each other condition. O: Oligomycin, F: FCCP, R&A: Rotenone and Antimycin A. **(F)** Two-week ALA 0.5μM pretreatment reduces cells' ability to increase ECAR in response to stressed conditions (oligomycin). *n*=6 replicates for Vehicle, *n*=5 replicates for each other condition. Data shown are mean +/- SEM. Data analyzed using Mann-Whitney test **(A, F),** ANOVA followed by Welch's correction **(C)**; $^{*}P < 0.05$, $^{**}P < 0.01$, $^{***}P < 0.001$.

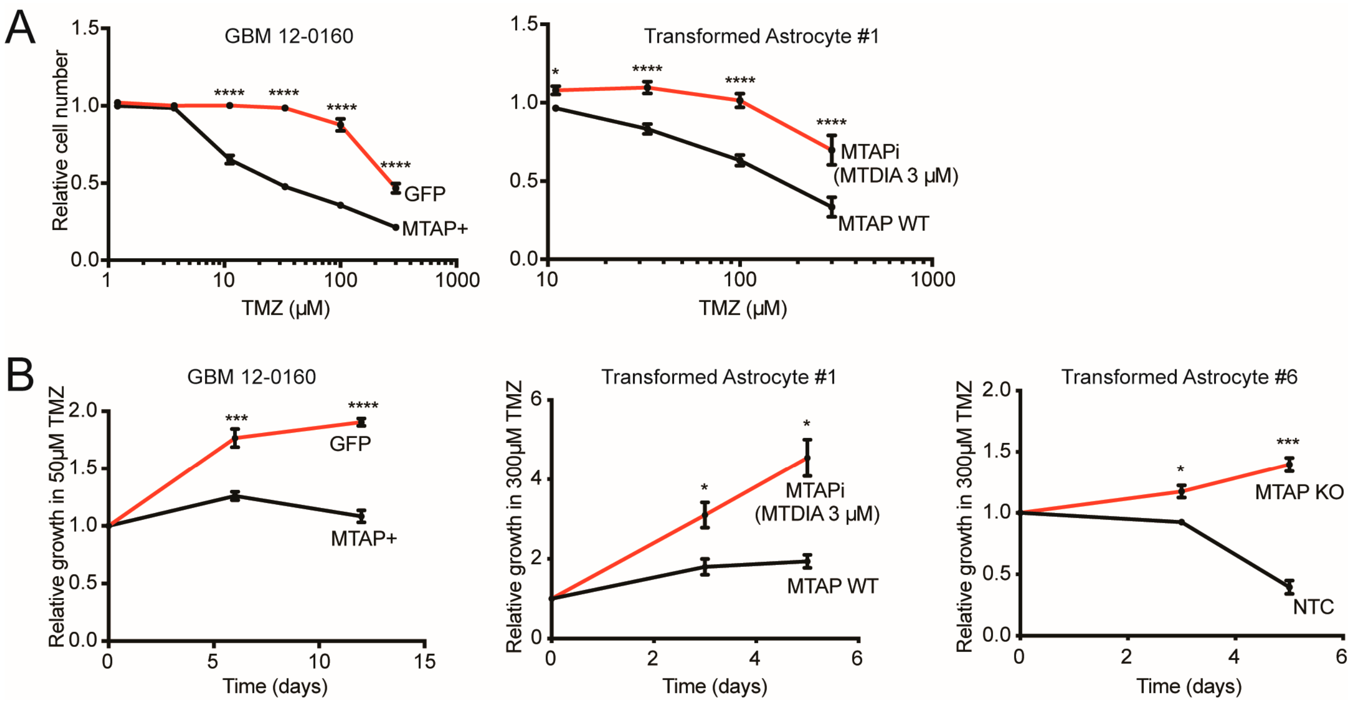

Figure S4. MTAP loss facilitates TMZ resistance.

(A) Drug sensitivity curves show GBM 12-0160 (left) and Transformed Astrocyte #1 (right) cells are more resistant to increasing doses of TMZ when MTAP is deficient (left) or pharmacologically inhibited (right). CCK8 assay, 4-6 days, *n*=4 replicates.

(B) Proliferation assays show genetic absence (left, right) or pharmacological inhibition (middle) of MTAP facilitate TMZ resistance in several GBM models. Manual cell counting using hemocytometer, *n*=3 replicates. Data shown are mean +/- SEM. Data analyzed using Two-Way ANOVA followed by Sidak's multiple comparisons test **(A)** or multiple unpaired t-tests **(B)**; *$P < 0.05$, ***$P < 0.001$, ****$P < 0.0001$.

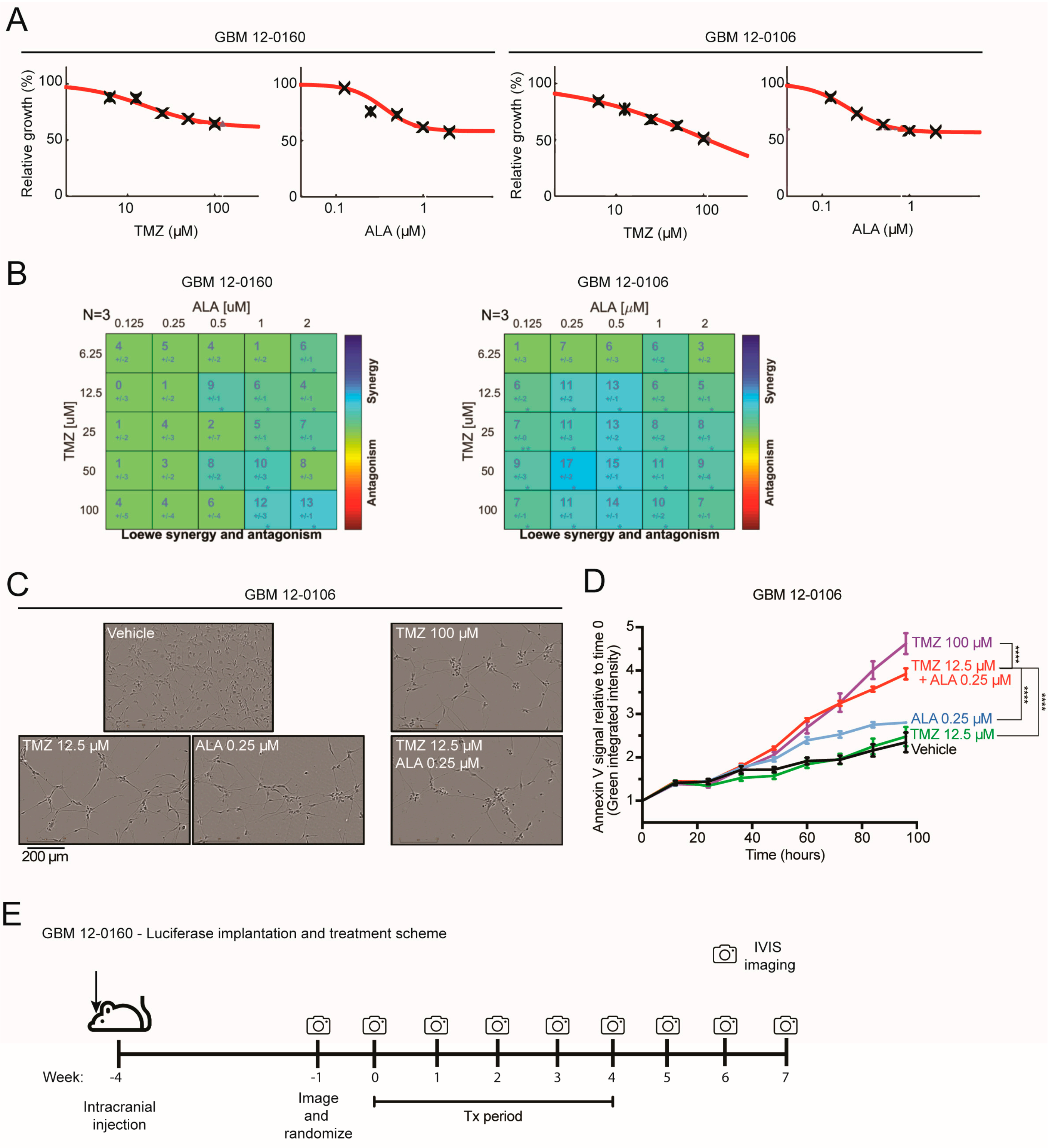

Figure S5. Synergy/antagonism and apoptosis analyses of TMZ + ALA combination treatment. **(A)** Single agent TMZ and ALA dose response curves for GBM cells. Plots generated by Combenefit software, mean shown of *n*=3 replicates. **(B)** Synergy/antagonism analysis of TMZ and ALA combination treatment data from Figure 3C. Each square indicates the synergy/antagonism score +/- SEM for a given dose combination after 4 days of treatment. Matrix plots generated by Combenefit

software, *n*=3 replicates for each drug combination. **(C)** Representative images of adherent GBM 12-0106. Images taken with Incucyte Live Cell Imaging system. Scale bar, 200μm. **(D)** Longitudinal Annexin V staining assay shows low-dose combination TMZ+ALA treatment leads to higher Annexin V signal than single agent treatment and similar signal as high-dose TMZ alone. **(E)** Scheme for *in vivo* drug response experiment using GBM 12-0160 – Luciferase. Data shown are mean +/- SEM. Data analyzed using Combenefit software **(B)**, Two-Way ANOVA followed by Tukey's multiple comparisons test **(D)**; $^{*}P < 0.05$, $^{**}P < 0.01$, $^{****}P < 0.0001$.
